# The Effectiveness of Laparoscopic-assisted Percutaneous Endoscopic Gastrostomy in Patients with Unfavorable Anatomy: A Single-center Retrospective Cohort Study

**DOI:** 10.7759/cureus.6647

**Published:** 2020-01-13

**Authors:** Daud Lodin, Anupam K Gupta, David Rubay, Thomas Genuit, Nir Hus

**Affiliations:** 1 Surgery, Charles E. Schmidt College of Medicine, Florida Atlantic University, Boca Raton, USA; 2 Surgery, Delray Medical Center, Delray Beach, USA

**Keywords:** percutaneous

## Abstract

Percutaneous endoscopic gastrostomy (PEG) is an easy means to provide enteral access in patients unable to maintain adequate nutrition via the oral route. In patients with morbid obesity, altered intra-abdominal anatomy due to prior abdominal surgery, the interposition of the colon or other factors precluding endoscopy, feeding tube placement by laparoscopic means (LAPEG) can provide a potentially safe alternative. The objective of this study was to examine the efficacy and outcomes of laparoscopic-assisted placement of PEG in adult patients. This is a retrospective cohort analysis of adult patients, who underwent PEG and/or laparoscopic-assisted percutaneous endoscopic gastrostomy placement (LAPEG) by two surgeons at a single institution. A total of 36 patients underwent PEG and/or LAPEG. No significant differences were found in the total and postoperative length of stay and mortality. There were no procedure-related complications in the LAPEG group versus two in the PEG group (8.7%), but this did not reach statistical significance. LAPEG was 100% successful in gaining enteral feeding access in patients that had failed PEG. The most common reason for PEG placement failure was colonic interposition (39%), followed by intra-abdominal adhesions and gastric displacement by hiatal hernia (each 23%). 38.5% of LAPG procedures could be done via 5-mm single port access, 38.5% required two-port and 23% required three-port access. In conclusion, LAPEG is a feasible minimally invasive alternative to gain enteral feeding access in patients that have failed PEG that does not increase the length of stay, morbidity or mortality.

## Introduction

First developed by Michael Gauderer and Jeffry Ponsky in 1980, percutaneous endoscopic gastrostomy (PEG) tubes have been in use for nearly 40 years as a convenient and minimally invasive method for access in patients who are unable to tolerate oral feeding for a variety of reasons [1]. However, at times, due to unfavorable body habitus or intrabdominal anatomy, PEG placement can be unsuccessful, and surgical placement of a feeding tube may be required.

In 1994, Raaf et al. published in the Journal of Laparoendoscopic Surgery, a novel method in which laparoscopy was used to aid in the placement of gastric feeding tubes in patients with unfavorable intraabdominal anatomy preventing simple endoscopic placement [2]. Since the article’s publication, several case studies have been published, detailing clinical scenarios in which the use of laparoscopic assistance may lead to successful placement of enteral feeding access, without the need for a more invasive procedure [3-8]. Much of the research on laparoscopically assisted PEG (LAPEG) placement has been focused on pediatric patient populations [9-12]. Only a few studies have examined larger cohorts of adult patients [13-14], and most of these studies originated in Japan [15-17]. Currently, there are no published series systematically comparing outcomes of LAPEG and PEG. This retrospective cohort study compares the outcomes of patients undergoing PEG and LAPEG in a North American population over a period of two years.

## Materials and methods

This is a single-institution, retrospective, cohort study examining the use of PEG and LAPEG in patients at a community-based tertiary care acute care hospital, from January 2016 to December 2018. Data were collected from patient health records, using the 2020 International Classification of Disease-10 (ICD-10) and American Medical Association Current Procedural Terminology (CPT) codes to identify PEG and enteral feeding tube placement in hospitalized patients. Open surgical gastrostomy and other feeding tube access procedures were excluded. Demographic information (age, gender, weight, BMI), procedure-specific data (use of laparoscopy, number and position of ports, necessity of adhesiolysis (LOA), reasons for the use of LAPEG, anatomical findings, and procedure-related complications) were recorded, and total length of stay (TLOS) and postoperative length of stay (POLOS) were collected to assess the impact of PEG and LAPEG on hospital stay and discharge. The data was kept using a secure database, and the OpenEpi web-based epidemiologic and statistical calculator was utilized to calculate statistical significance, using chi-square, analysis of variance (ANOVA), and T-testing. A p-value of <0.05 was utilized to determine statistical significance.

Surgical decision and technique for LAPEG

**Figure 1:**
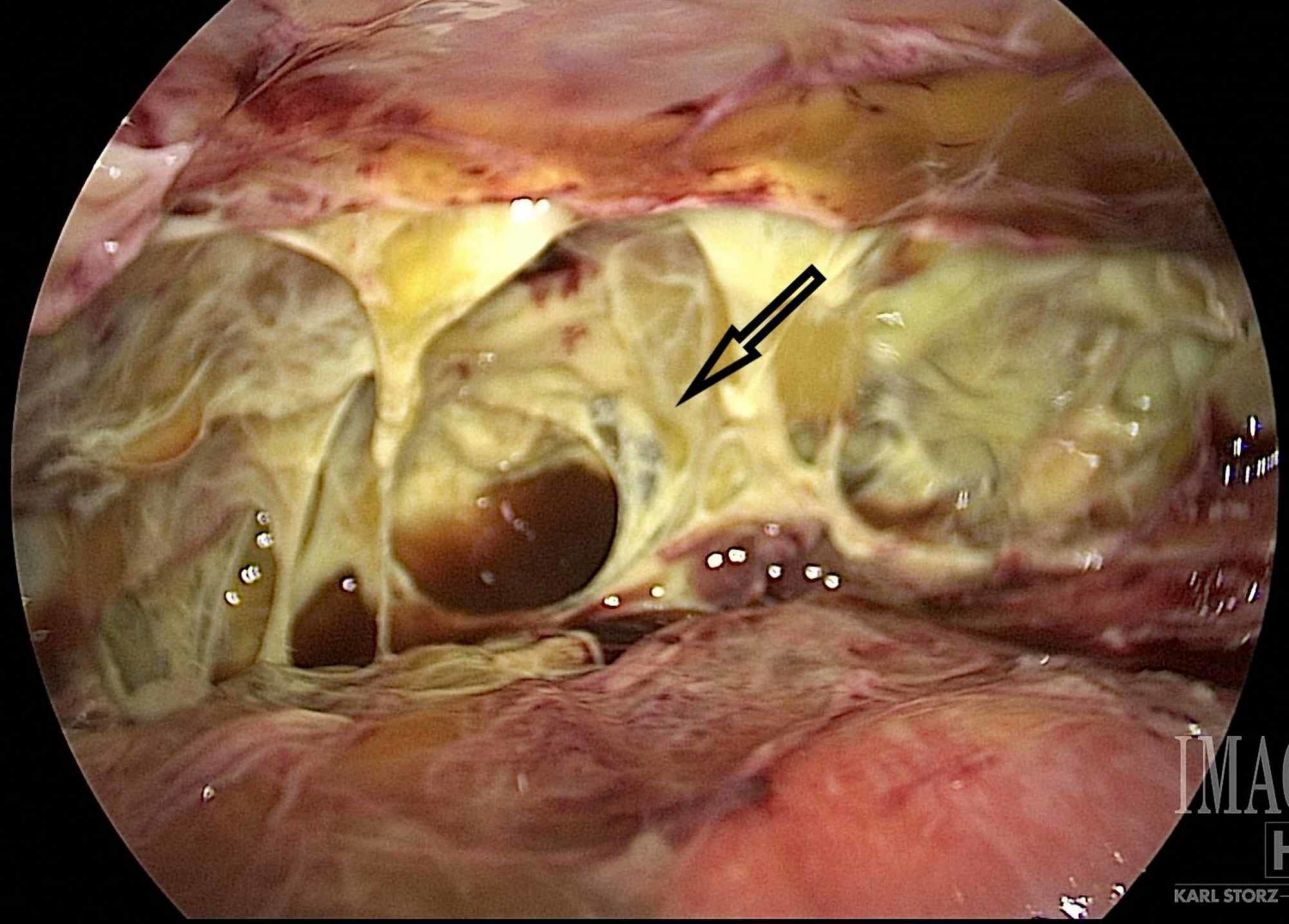
Initial inspection of a difficult abdomen containing several adhesions and abscess fluid.

Patients who failed PEG placement was selected to undergo LAPEG placement. Failed PEG placement was defined as the inability to place the feeding tube endoscopically or radiologically, using generally accepted safety standards for these procedures or tube malfunction within 24-48 hours after placement [18]. A standardized surgical approach was utilized. Patients underwent general anesthesia and were placed in a supine position. A baseboard was installed at the foot-end of the operating table to facilitate steep reverse Trendelenburg position when needed. A 5-mm peri-umbilical port and a zero-degree optic camera were utilized. Initial pneumoperitoneum was limited to 5 mm Hg. A diagnostic laparoscopy was performed, focused on the upper abdomen, looking for adhesions, the position of the stomach, and any other intraabdominal findings that would prevent gastrostomy tube placement (Figure 1).

**Figure 2:**
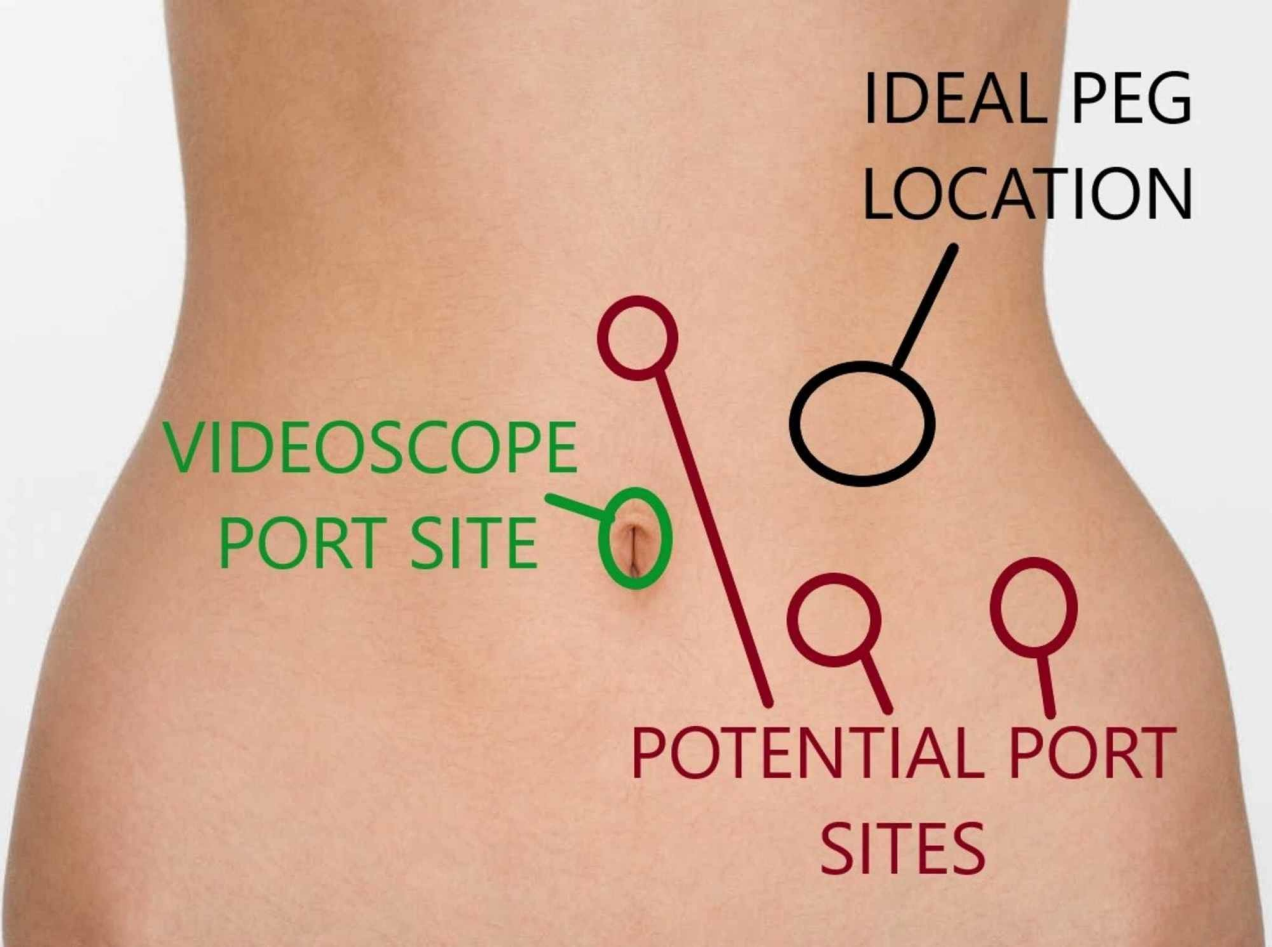
The locations and placement of videoscope or additional laparoscopic ports to assist in percutaneous endoscopic gastrostomy tube placement.

For lysis of adhesions, insufflation pressures were raised to 12-15 mm Hg. Patients were placed in a reverse Trendelenburg position to allow for downward displacement of the transverse colon and facilitate the apposition of the stomach to the anterior abdominal wall. Up to two additional 5-mm laparoscopic ports were placed to assist in the procedure, as needed (Figure 2).

**Figure 3:**
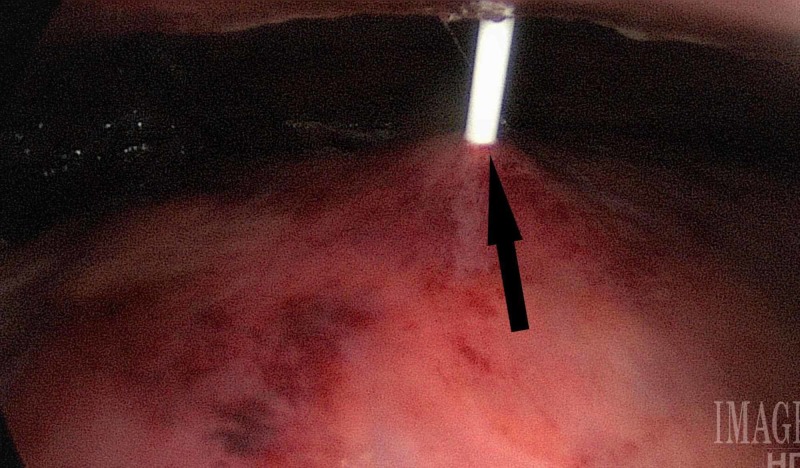
Direct visualization of the percutaneous endoscopic gastrostomy needle entering passing through the skin and into the stomach. The arrow indicates successfully placement of the gastrostomy.

Gastrostomy tube placement began with the insertion of the gastroscope into the stomach via the oral route. The abdomen was insufflated to allow approximation of the anterior gastric wall, anterior abdominal wall. A 16-gauge needle was inserted into the stomach through the abdominal wall, and the pull-through method was used to place the gastrostomy tube under laparoscopic visualization (Figure 3).

Upon completion, the abdomen was deflated, and the ports were removed under direct visualization. Skin incisions were closed primarily. Following the procedure, the gastrostomy function was confirmed with flushing and aspiration of saline. Feeding and administration of medications were resumed on the day of the procedure.

## Results

Thirty-six patients underwent attempted PEG placement during the study time period. In 13 patients, endoscopic or radiologic PEG placement was unsuccessful and these patients underwent LAPEG. No statistically significant differences were noted in patient demographics (Table 1) for either groups including age (mean 68.8 versus 62.4 years, p = 0.36), gender (17.4 versus 23.1% female, p = 0.68), body-mass index (BMI) (mean 26.4 versus 24.8 kg/m^2^, p = 0.50), or BMI greater than 35 kg/m^2^ (13.0 versus 15.4 percent, p = 0.85). There were no differences in the indication for feeding tube placement in patients that underwent successful and unsuccessful PEG placement. The LAPEG group appeared to have a slightly increased incidence of pre-existing (longer-term) dysphagia (53.8% LAPEG versus 39.1%), whereas patients with successful PEG placement had more acute dysphagia (56.5% PEG versus 30.8% LAPEG), often related to prolonged intubation, but this did not reach statistical significance.

**Table 1:** Demographics, length of stay, complications, and medical indications of the need for gastrotomy in patients that underwent standard or laparoscopic-assisted percutaneous gastrostomy tube placement. BMI: Body mass index; TLOS: Total length of stay; POLOS: Postoperative length of stay; STD: Standard deviation.

		PEG	LAPEG		
	(N or Mean)	(% or STD)	(N or Mean)	(% or STD)	P
n	23	63.9%	13	36.1%	
Female	4	17.4%	3	23.1%	0.68
BMI (kg/m^2^)	26.4	7.5	24.8	5.3	0.50
BMI ≥ 35 kg/m^2^	3	13.0%	2	15.4%	0.85
Age (Years)	68.8	17.2	62.4	20.9	0.36
TLOS (Days)	33.0	28.8	26.0	13	0.42
POLOS (Days)	22.4	29.7	10.8	9.3	0.19
Complications	2	8.7%	0	0.0%	0.28
INDICATION FOR G-TUBE					
Dysphagia	9	39.1%	7	53.8%	
Prolonged Intubation	13	56.5%	4	30.8%	
Other	1	4.3%	2	15.4%	0.25

Average TLOS between PEG (33.0 days) and LAPEG (26.0 days) patients showed no statistically significant difference (p = 0.42). Average POLOS tended to be shorter in LAPEG (10.8 days) patients versus PEG patients (22.4 days), but this was statistically insignificant (p = 0.19). Only two procedure-related complications were noted, both arising in the PEG group. One patient suffered cardiac arrest during PEG placement and another patient suffered a gastric injury that needed surgical repair.

The most common intraoperatively identified reason for failed PEG placement was colonic interpositions (five of 13, 38.5%). 23.1% of patients (three of 13) had significant adhesions, 23.1% demonstrated gastric displacement by hiatal hernia and 15.4% (two of 13) had other intra-abdominal inflammatory processes (Table 2). The lysis of adhesions was required in 30.8% of patients (four of 13). 38.5% of patients (five of 13) required only one 5-mm peri-umbilical port, 38.5% required two ports, and the remainder of patients required three ports. All LAPEG-placed tubes were fully functional immediately after the procedure and did not have any complications or malfunctions to the day of discharge. No patient required conversion to an open gastrotomy tube placement.

**Table 2:** Surgical indications, number of ports, and need for lysis of adhesion for patients that underwent laparoscopic-assisted percutaneous gastrostomy tube placement.

	n	%
Transverse Colon Preventing Access	5	38.5%
Presence of Adhesions or Abscess	3	23.1%
Hiatal Hernia or Elevated Stomach	3	23.1%
Infection or Perforated Stomach	2	15.4%
One Port (for Visualization)	5	38.5%
Two Ports (Visualization & Assistance)	5	38.5%
Three Port (Visualization & Assistance)	3	23.1%
Number that Required Adhesion Lysis	4	30.8%

## Discussion

For patients that require enteric nutrition but are unable to tolerate oral feeding, the advent of minimally invasive placement of enteric feeding tubes revolutionized patient care. In those patients with difficult intraabdominal anatomy, laparoscopic assistance is a feasible adjunct to endoscopic and radiologic placement, when those modalities fail. This study demonstrates that LAPEG does not significantly add to the length of stay or rate of complications. The success rate was 100%, and tubes were safely used immediately after the procedure. Due to the small size of our study, none of our study outcomes reached statistical significance, but there was a trend to shorter POLOS and fewer procedure-related complications in the LAPEG group. Direct visualization of the anterior gastric wall and tube insertion site allows for avoidance of areas of gastric friability, visible vessels in the stomach, or anterior abdominal wall, which should reduce complications (Figure 3). While there were no significant differences in BMI between study groups and overall BMI was relatively low, in a follow-up study, we aim to evaluate the feasibility of LAPEG in high BMI patients. As described by McGarr and Kirby, in a study on PEG placement in obese patients, the lack of transillumination and finger indentation may prevent successful PEG placement even in the absence of intra-abdominal obstacles [19].

In most cases, this is due to colon interposition, a condition that can easily be addressed by placing the patient in steep reverse Trendelenburg position and gently manipulating the colon with the camera. This positioning is often not possible in the endoscopy or IR suite and requires the surgeon foresight to place a baseboard at the foot-end of the operating room table [20]. Through the addition of a second or third port, LAPEG allows the surgeon to address a variety of intra-abdominal findings that could preclude PEG placement. No complications were encountered due to additional port placement.

Study limitations

Due to the small study size, lack of case matching, and the retrospective nature of this study, outcomes did not reach statistical significance. The study did not include enough high-BMI patients to draw conclusions on this subset of patients. No assertions can be made on the long-term functionality of the PEG versus LAPEG tubes or delayed occurrence of complications. Future investigation is needed to address these issues and procedure-related costs.

## Conclusions

LAPEG is a feasible minimally invasive alternative to gain enteral feeding access in patients that have failed PEG. It does not increase the length of stay, morbidity, or mortality. Surgeons trained in laparoscopic procedures should be able to perform this procedure with minimal difficulty and risk of complications.
